# Reliability of TMS measurements using conventional hand-hold method with different numbers of stimuli for tibialis anterior muscle in healthy adults

**DOI:** 10.3389/fncir.2022.986669

**Published:** 2022-09-30

**Authors:** Bin Su, Yanbing Jia, Li Zhang, Duo Li, Qianqian Shen, Chun Wang, Yating Chen, Fanglan Gao, Jing Wei, Guilan Huang, Hao Liu, Lin Wang

**Affiliations:** ^1^School of Exercise and Health, Shanghai University of Sport, Shanghai, China; ^2^Department of Rehabilitation, Wuxi Central Rehabilitation Hospital, Wuxi, China; ^3^School of Rehabilitation Medicine, Jiangsu Vocational College of Medicine, Yancheng, China; ^4^Neuro-Rehabilitation Center, JORU Rehabilitation Hospital, Yixing, China

**Keywords:** transcranial magnetic stimulation, corticomotor excitability, tibialis anterior muscle, healthy adults, reliability

## Abstract

**Objective:** The objective of this study was to determine the reliability of corticomotor excitability measurements using the conventional hand-hold transcranial magnetic stimulation (TMS) method for the tibialis anterior (TA) muscle in healthy adults and the number of stimuli required for reliable assessment.

**Methods:** Forty healthy adults participated in three repeated sessions of corticomotor excitability assessment in terms of resting motor threshold (rMT), slope of recruitment curve (RC), peak motor evoked potential amplitude (pMEP), and MEP latency using conventional TMS method. The first two sessions were conducted with a rest interval of 1 h, and the last session was conducted 7–10 days afterward. With the exception of rMT, the other three outcomes measure elicited with the block of first 3–10 stimuli were analyzed respectively. The within-day (session 1 vs. 2) and between-day (session 1 vs. 3) reliability for all four outcome measures were assessed using intraclass correlation coefficient (ICC), standard error of measurement, and minimum detectable difference at 95% confidence interval.

**Results:** Good to excellent within-day and between-day reliability was found for TMS-induced outcome measures examined using 10 stimuli (ICC ≥ 0.823), except in pMEP, which showed between-day reliability at moderate level (ICC = 0.730). The number of three stimuli was adequate to achieve minimum acceptable within-day reliability for all TMS-induced parameters and between-day reliability for MEP latency. With regard to between-day reliability of RC slope and pMEP, at least seven and nine stimuli were recommended respectively.

**Conclusion:** Our findings indicated the high reliability of corticomotor excitability measurement by TMS with adequate number of stimuli for the TA muscle in healthy adults. This result should be interpreted with caveats for the specific methodological choices, equipment setting, and the characteristics of the sample in the current study.

**Clinical Trial Registration:** http://www.chictr.org.cn, identifier ChiCTR2100045141.

## Introduction

Transcranial magnetic stimulation (TMS) is a non-invasive technique (Barker et al., 1985) and has been widely used in evaluating brain plasticity (Hallett, 1996; Rothwell, 2011). According to the principle of electromagnetic induction, magnetic stimulation acts on the primary motor cortex of the brain (M1) and induces neural axon depolarization, which can be recorded in corresponding peripheral muscle with surface electromyography; the detected signal is called motor-evoked potential (MEP; Rotenberg et al., 2014; Rossini et al., 2015). Hence, MEP and its related parameters induced by TMS can certainly reflect the excitability of cortico-spinal pathway and are commonly used in neurophysiological examination and research (Moscatelli et al., 2021).

Changes in measurements should reflect actual changes in patients’ conditions rather than those caused by measurement errors. Therefore, testing the reliability of an assessment tool should be the primary consideration in clinical and research; unreliable measurements are prone to large systematic and random errors (Atkinson and Nevill, 1998). TMS-related outcome measures are variable, and thus their reliability has attracted considerable interest, that is, TMS can provide accurate and consistent measurements for individuals without physiological changes. Although some new techniques such as neuronavigation, robot TMS coil holding arm, and EEG-TMS closed-loop triggering strategy which could improve the accuracy of hotspot localization and reduce the variability of TMS-induced MEP (Goetz et al., 2019), these advanced techniques are more widely applied in some high-level lab for research studies. For clinical evaluation, the conventional methods of TMS using hand-hold assessment are still used by most medical institutions. On the other hand, different shapes of coil can be used for TMS assessment. Although the double-cone coils have been around for decades, there is as yet few evidence of the reliability of lower limb assessment with double-cone coils compared to upper limb studies and numerous coil brands (Beaulieu et al., 2017a).

MEP-related parameters considerably vary with the number of stimuli delivered per site during TMS assessment (Kiers et al., 1993; Wassermann, 2002). Increasing the number of stimuli may reduce the influence of inherent variations in an individual (Bastani and Jaberzadeh, 2012). However, this approach brings another problem, that is, more testing time is needed. Prolonged TMS tests would make participants feel uncomfortable. Therefore, the minimum number of stimuli to obtain reliable MEP parameters must be investigated. For reliable MEP, a minimum of five stimuli are recommended for within-session comparisons, and 10 stimuli are recommended for between-session comparisons (Cavaleri et al., 2017). Lewis et al. (2014) demonstrated that at least six magnetic stimuli are required for the reliable MEP of the soleus muscle in the same session of stroke assessment, whereas the intersession reliability was poor even with ten stimuli applied for average MEP assessment. However, studies on the assessment of lower limbs subjected to different numbers of stimuli have mainly focused on MEP amplitude, and reports on the slope of recruitment curve (RC) and MEP latency are few (Lewis et al., 2014).

With respect to the foregoing, the purpose of this study was to investigate the reliability of TMS-induced parameters in measuring the cortico-spinal excitability of the lower limb using a double-cone coil and conventional hand-hold method, and determine the number of TMS stimuli required to achieve acceptable reliable measurement. The results of the present study would provide an evidence-based knowledge about TMS as a tool for evaluating the cortico-spinal excitability of the lower limb in clinical practice and research.

## Materials and Methods

### Participants

Forty healthy volunteers aged 19–36 years (23.05 ± 3.83 years; 21 males) were recruited using an advertising poster from January 1, 2021 to August 31, 2021. According to the safety guidelines of TMS (Rossi et al., 2021), all participants were screened for the contraindications of TMS. The inclusion criteria were as follows: (i) age ≥ 18 years; (ii) no central nervous system lesion; (iii) no serious mental or physical disease; and (iv) no drug or alcohol dependence history; and (v) no contraindications to TMS examinations. The exclusion criteria were as follows: (i) any neurological disease; (ii) history of seizures; (iii) previous adverse effects with TMS; (iv) taking medications that affect cortical excitability; (v) pregnancy; (vi) history of lower limb surgery; and (vii) poor compliance or failure to cooperate during testing. Participants were asked to have a good sleep the night before the test, avoid taking recreational drugs or alcohol on the day before the test and avoid drinking caffeinated drinks 2 h before the test.

### Experimental design and measurement procedures

This study adopted a repeated-measures design and was approved by the Research Ethics Committee of the JORU Rehabilitation Hospital (No. 20210325B02, ChiCTR2100045141). After signing informed consent, all participants underwent three sessions of assessment on corticomotor excitability with TMS for the tibialis anterior (TA) muscles of the non-dominant leg. The dominant legs of the participants were defined as the legs they would use to kick a ball (van Melick et al., 2017). The first two sessions were conducted on the first day, with a rest interval of 1 h. The last session was conducted 7–10 days afterward at the same time of the day as the first session. For each participant, all three assessment sessions were performed from 15:00 to 18:00 of the testing day by the same rater, who was a physical therapist with extensive experience on corticomotor excitability measurement using TMS.

The strength of a magnetic field induced by TMS is attenuated as the distance from the scalp surface increases (Rothwell et al., 1991; Deng et al., 2013). However, the M1 areas of the lower limb are located at the paracentral lobules, which are deeper than those of the upper limb (Rothwell et al., 1991; Rossini et al., 2015). Therefore, an electric field induced by TMS using figure-of-eight or round coil may be inadequate to activate motor neurons that control the muscles of the lower limbs (Dharmadasa et al., 2019). The double-cone coil, a figure-of-eight-shaped coil with two components at each angle, increases the strength of a magnetic field (Hallett, 2007). The magnetic field penetration depth of a double-cone coil is significantly greater than that of a figure-of-eight coil (Deng et al., 2013, 2014; Lu and Ueno, 2017). Thus, a double-cone coil stimulates the M1 region of the lower limbs more easily and is the recommended tool for examining the neurophysiological status involving lower limbs. Hence, in the present study, the corticomotor excitability measurement for the TA muscle of the non-dominant leg was measured with a Magneuro100 stimulator and a matching double-cone coil (VCZ001; VISHEE Company Limited, Nanjing, China).

During the assessment, each participant sat comfortably upright in a high back chair, and the arms, back, and legs supported. After skin preparation, a pair of silica gel electrodes was placed over the belly of the TA muscle at the upper third of the lower leg. The distance between the electrodes was 2 cm. A ground electrode was applied ipsilaterally on the lateral malleolus. Electromyography (EMG) signals were amplified (gain 1,000×), filtered (bandpass: 20–500 Hz), and sampled at 2,000 Hz with a wireless portable motor evoked potential detection module. During the measurement, participants were instructed to stay relaxed, and the relaxed state was monitored through the visual inspection of the EMG recordings in real time. When obvious muscle activities were detected, the participants were reminded, and the procedure was suspended until the muscle relaxes again.

**Figure 1:**
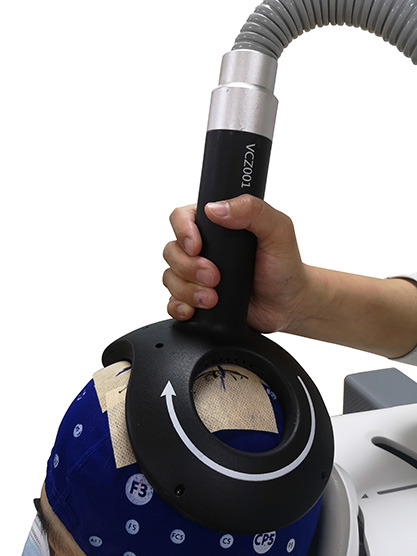
Coil handle is perpendicular to the scalp.

During the measurements, a positioning cap designed according to the international 10–20 EEG system was fixed on a participant’s head. Biphasic single magnetic pulses were generated at least every 5 s to stimulate the M1 area contralateral to the recorded TA muscle around the Cz point, and the coil handle was perpendicular to the scalp (Farzan, 2014; Figure 1). The current direction in the long axis of the two-wing intersection of the coil was produced at AP orientation which would induce a PA oriented current in the cortex of the lower limb (Groppa et al., 2012). The optimal stimulation site, which was called the “hotspot”, for TA muscle was identified by moving the double-cone coil in 1 cm steps at the initial stimulation intensity and a supposed suprathreshold level and marked using a pen as the easiest excitable site that can consistently elicit large MEP amplitudes at a relatively low TMS output intensity. For each session, the same procedure used for hotspot identification was conducted prior to the corticomotor excitability outcome measurement. After the hotspot for TA muscle was identified, the resting motor threshold (rMT) was determined with the TMS coil fixed on the hotspot, and the suprathreshold stimulator output intensity was reduced by 2% stepwise, and then by 1% when the intensity was near the threshold intensity. The rMT is the lowest TMS output intensity that can induce MEP amplitudes above 0.05 mV in at least five of 10 consecutive TMS stimuli when a TMS coil is fixed on the hotspot of the TA muscle (Liu and Au-Yeung, 2014). The MEPs were measured using TMS output intensities ranging from 90% rMT to 160% rMT, and the intensity was increased by 10% for every 10 stimuli. RCs were plotted in Microsoft Excel with the MEP amplitude averaged using different numbers of consecutive MEPs from the first three to ten evoked at the same intensity against a corresponding TMS output intensity level. The RC slope was obtained by adding the linear regression line to the curve (Liu and Au-Yeung, 2014). The MEP latency was defined as the time from the delivery of TMS stimuli to MEP onset which was identified by the cursor of the self-contained system of motor evoked potential detection module and inspected visually by an investigator.

The rMT, linear slope for RC, maximum mean MEP amplitudes that were reached during RC measurement (pMEP), and mean MEP latency evoked at 130% rMT were adopted as the outcome measures for the reliability test.

### Data analysis and statistics

Data analyses were performed by using the software package of SPSS23.0. Demographic data were statistically described with means ± standard deviations to represent the characteristics of the participants. Within-day reliability (session 1 vs. 2) and between-day reliability (session 1 vs. 3) were evaluated using intraclass correlation coefficients (ICCs), and their 95% confidence interval limit was based on a two-way mix model (Model 3). The level of significance was set at 0.05. In the reliability analysis, the MEP latencies evoked by 130% rMT were averaged for 3–10 stimuli, whereas the RC slopes plotted from 90% rMT to 160% rMT were calculated for 3–10 stimuli delivered at each of the series of intensities. The pMEPs were the maximum MEP amplitudes during the RC procedure at different numbers of stimuli. The reliability of the measurements was assessed as follows: >0.90, excellent; 0.75–0.90, good; 0.50–0.75, moderate; and <0.50, poor (Portney and Watkins, 2009).

According to the reliability coefficient, the standard error of measurement [SEM = (pooled SD) × √(1-ICCs)] and the minimum detectable measurement difference (MDD_95_ = 1.96 × SEM × √2) were calculated for the analysis of response stability and test responsiveness of TMS-induced corticomotor excitability. The SEM showed variation around a “true” score of excitability parameters for an individual when repeated measures were taken. The reliability of the measurement results increased with decreasing SEM value. The MDD_95_ represents the smallest difference value, which can be confirmed as the “true change” in cortical motor excitability measurement values within the 95% confidence interval.

## Results

**Figure 2:**
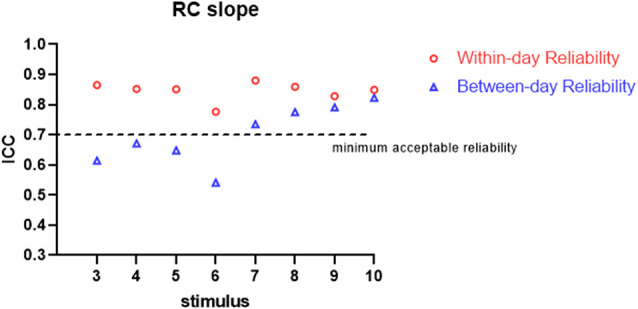
Within-day and between-day reliability of RC slope. ICC, intraclass correlation coefficient.

**Figure 3:**
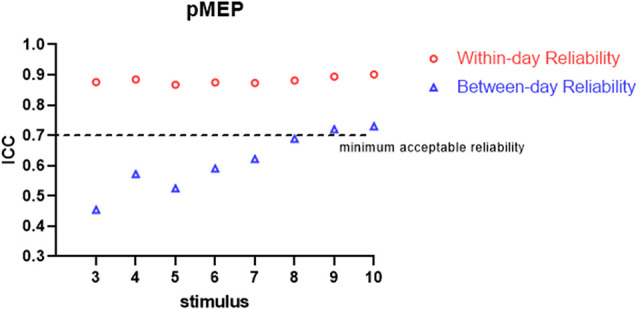
Within-day and between-day reliability of pMEP. ICC, intraclass correlation coefficient.

**Figure 4:**
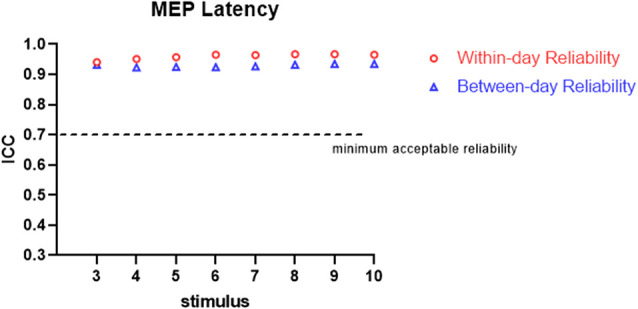
Within-day and between-day reliability of MEP latency. ICC, intraclass correlation.

All the participants completed the whole procedure of the study. The demographic characteristics of all participants are presented in Table 1. Table 2 lists the reliability analysis in terms of the ICC, SEM, and MDD_95_ of the four TMS-induced outcome measures related to cortical excitability. The within-day comparison showed excellent reliability for rMT (ICC = 0.946, *p* < 0.001) and MEP latency (ICC ≥ 0.941; 3–10 MEPs; *p* < 0.001), good reliability for RC slope (ICC = 0.776–0.880; 3–10 MEPs; *p* < 0.001), and good to excellent reliability for pMEP (ICC = 0.867–0.901; 3–10 MEPs; *p* < 0.001). With regard to between-day reliability, the rMT (ICC = 0.936, *p* < 0.001) and latency (ICC ≥ 0.923; 3–10 MEPs; *p* < 0.001) displayed excellent reliability. However, the ICCs revealed moderate to good reliability for the RC slope (ICC = 0.541–0.823; 3–10 MEPs; *p* < 0.01) and poor to moderate reliability for pMEP (ICC = 0.454–0.730; 3–10 MEPs; *p* < 0.05). When the number of stimuli was greater than 7 and 9 for the repeated assessment on different days, the ICC values of the RC slope (ICC = 0.735) and pMEP (ICC = 0.720) achieved values higher than 0.7 respectively, which is defined as the minimum acceptable reliability for outcome measures (Matheson, 2019; Figures 2–4).

**Table 1:** Characteristics of participants.

**General characteristics (*n* = 40)**	
Gender (n)	21/19
Age (years)	23.05 ± 3.83–36
Height (cm)	167.70 ± 7.75
Weight (kg)	60.96 ± 9.64
foot dominance (n)	35/5

n = number.

**Table 2:** Within-day and between-day reliability measures for the participants.

**Outcomes (n = 40)**		**S1 (mean ± SD)**	**S2 (mean ± SD)**	**S3 (mean ± SD)**	**Within-day (S_1_ vs S_2_)**					**Between-day (S_1_ vs S_3_**				
					**ICC**	**95%CI**	***p***	**SEM**	**MDD_95_**	**ICC**	**95%CI**	***p***	**SEM**	**MDD_95_**
rMT (% MSO)		39.875 ± 11.209	38.825 ± 10.135	39.325 ± 10.545	0.946	0.898–0.971	<0.001	2.483	6.883	0.936	0.879–0.966	<0.001	2.753	7.631
RC slope	3MEPs	0.029 ± 0.026	0.027 ± 0.021	0.022 ± 0.019	0.865	0.745–0.929	<0.001	0.009	0.024	0.614	0.270–0.796	0.002	0.014	0.039
	4MEPs	0.029 ± 0.025	0.026 ± 0.021	0.022 ± 0.019	0.852	0.720–0.922	<0.001	0.009	0.025	0.671	0.377–0.826	<0.001	0.013	0.035
	5MEPs	0.029 ± 0.026	0.026 ± 0.020	0.022 ± 0.019	0.851	0.718–0.921	<0.001	0.009	0.025	0.648	0.334–0.814	0.001	0.014	0.037
	6MEPs	0.031 ± 0.028	0.026 ± 0.020	0.021 ± 0.018	0.776	0.577–0.882	<0.001	0.012	0.032	0.541	0.132–0.757	0.008	0.016	0.044
	7MEPs	0.028 ± 0.023	0.027 ± 0.020	0.021 ± 0.018	0.880	0.774–0.937	<0.001	0.007	0.021	0.735	0.499–0.860	<0.001	0.011	0.029
	8MEPs	0.028 ± 0.022	0.028 ± 0.020	0.021 ± 0.018	0.859	0.732–0.925	<0.001	0.008	0.022	0.775	0.575–0.881	<0.001	0.010	0.026
	9MEPs	0.027 ± 0.022	0.029 ± 0.025	0.022 ± 0.017	0.828	0.675–0.909	<0.001	0.010	0.027	0.791	0.606–0.890	<0.001	0.009	0.025
	10MEPs	0.027 ± 0.021	0.029 ± 0.026	0.022 ± 0.017	0.849	0.714–0.920	<0.001	0.009	0.025	0.823	0.665–0.906	<0.001	0.008	0.022
(pMEPμv)	3MEPs	275.073 ± 218.254	233.633 ± 154.162	240.688 ± 146.837	0.876	0.765–0.934	<0.001	66.534	184.424	0.454	−0.032–0.711	0.031	137.443	380.972
	4MEPs	266.243 ± 203.872	229.370 ± 146.878	231.015 ± 138.796	0.885	0.783–0.939	<0.001	60.253	167.012	0.572	0.191–0.774	0.005	114.093	316.250
	5MEPs	262.218 ± 214.974	230.090 ± 146.874	229.950 ± 141.036	0.867	0.748–0.930	<0.001	67.140	186.102	0.525	0.101–0.749	0.011	125.299	347.312
	6MEPs	257.068 ± 206.168	231.523 ± 147.218	223.093 ± 137.575	0.875	0.763–0.934	<0.001	63.334	175.552	0.591	0.226–0.783	0.003	112.084	310.682
	7MEPs	252.838 ± 196.868	231.533 ± 146.661	218.600 ± 130.454	0.873	0.759–0.933	<0.001	61.862	171.473	0.622	0.286–0.800	0.002	102.672	284.592
	8MEPs	250.515 ± 189.464	233.983 ± 150.439	214.718 ± 129.882	0.881	0.775–0.937	<0.001	59.012	163.574	0.689	0.412–0.836	<0.001	90.582	251.081
	9MEPs	246.570 ± 180.875	232.560 ± 151.272	212.250 ± 127.076	0.894	0.800–0.944	<0.001	54.284	150.467	0.720	0.470–0.852	<0.001	82.710	229.261
	10MEPs	242.203 ± 174.372	232.610 ± 151.329	208.748 ± 126.233	0.901	0.813–0.948	<0.001	51.368	142.384	0.730	0.490–0.857	<0.001	79.095	219.239
Latency	3MEPs	28.373 ± 1.701	28.393 ± 1.852	28.493 ± 1.569	0.941	0.888–0.969	<0.001	0.432	1.197	0.932	0.872–0.964	<0.001	0.427	1.183
	4MEPs	28.415 ± 1.660	28.395 ± 1.854	28.513 ± 1.572	0.951	0.907–0.974	<0.001	0.390	1.080	0.923	0.855–0.959	<0.001	0.449	1.243
	5MEPs	28.435 ± 1.662	28.425 ± 1.825	28.490 ± 1.587	0.957	0.919–0.977	<0.001	0.362	1.003	0.925	0.859–0.960	<0.001	0.445	1.233
	6MEPs	28.460 ± 1.685	28.423 ± 1.829	28.495 ± 1.581	0.965	0.934–0.982	<0.001	0.329	0.912	0.924	0.857–0.960	<0.001	0.450	1.248
	7MEPs	28.448 ± 1.681	28.415 ± 1.865	28.463 ± 1.592	0.964	0.933–0.981	<0.001	0.337	0.934	0.927	0.861–0.961	<0.001	0.442	1.226
	8MEPs	28.445 ± 1.657	28.395 ± 1.845	28.455 ± 1.603	0.967	0.938–0.983	<0.001	0.319	0.883	0.932	0.872–0.964	<0.001	0.425	1.178
	9MEPs	28.425 ± 1.662	28.385 ± 1.859	28.453 ± 1.605	0.967	0.938–0.983	<0.001	0.320	0.888	0.935	0.877–0.966	<0.001	0.417	1.155
	10MEPs	28.440 ± 1.644	28.400 ± 1.871	28.483 ± 1.587	0.965	0.933–0.981	<0.001	0.329	0.913	0.935	0.878–0.966	<0.001	0.412	1.142

rMT, resting motor threshold; % MSO, percentage maximum stimulator output; RC, recruitment curve; MEP, motor evoked potential; pMEP, peak MEP amplitudes; S1, session 1; S2, session 2; S3, session 3; SD, standard deviation; ICC, intraclass correlation coefficient; CI, confidence interval; SEM, standard error of measurement; MDD_95_, minimum detectable difference based on a 95% confidence interval.

## Discussion

In this study, we assessed the within-day and between-day reliability of TMS-related outcome measures induced by various numbers of stimuli. A double-cone coil was used for the TA muscle of the non-dominant lower limb of healthy young participants. The results demonstrated good to excellent within-day and between-day repeatability for all measurements examined by 10 stimuli except pMEP which showed moderate between-day reliability. Three stimuli were sufficient to achieve minimum acceptable within-day reliability for all TMS-induced parameters and between-day reliability for MEP latency. Regarding between-day reliability of the RC slope and pMEP, a minimum of seven and nine stimuli were recommended, respectively.

Early reports suggested that ICC, SEM, and MDD_95_ are the most accurate indexes in reliability studies (Weir, 2005; Beaulieu et al., 2017a; Lewis et al., 2020). ICC was recommended as the most suitable statistical test method for reliability parameter as it reflects the correlation and consistency of the results among repeated measurements (Portney and Watkins, 2000). SEM determines the degree of variation of measurements in a sample of individuals (Atkinson and Nevill, 1998). The accuracy of measurement and the sensitivity to changes increases with decreasing measurement error. Understanding the measurement error associated with TMS-induced outcome measures provides the profile of TMS as a tool for assessing corticomotor excitability. Notably, the MDD_95_ value for TMS-related parameters achieved in the present study can be used in determining the true change in the corticomotor excitability of the lower limb in a population of individuals with similar characteristics.

The ICC values for the repeatability of all TMS-related parameters in the between-day tests were lower than those of the within-day in the present study. This finding was coherent with the previous findings of studies, which have demonstrated that the MDD values of TMS-induced outcome measures when a test was repeated on the same day were lower than those in tests performed several days apart regardless of whether the test was conducted in the upper limb muscles or the lower limb muscles (Cacchio et al., 2009; Bastani and Jaberzadeh, 2012; Fisher et al., 2013). This result may be attributed to the larger time-variant neurophysiological fluctuations in the cortex on different days as compared with those on the same day (Wassermann, 2002). In addition, although hotspot measurements were repeated for each session, the EEG cap was not removed between session 1 vs. 2. Re-wearing the EEG cap in session 3 may have led to slight differences in the positioning of the hotspot and placement of the double-cone coil between measurements even when the international EEG 10–20 system was used in the alignment. Similarly, the difference in the placement of EMG electrodes between 2 days might have produced a bias in the results on MEP amplitudes. These methodological factors might also cause the lower reliability of between-day tests than the within-day tests.

The TMS-related parameters in the present study were recommended to reflect the different aspects of corticomotor excitability. rMT was defined as the lowest output intensity of TMS to induce the depolarization of neural axons and can reflect the excitability of the membrane of motor neurons. The RC slope was suggested to illustrate the degree of recruitment of the corticospinal pathway during stimulation with graded intensities (Devanne et al., 1997), whereas the pMEP amplitude reflects the maximum descending responses from the corticomotor neurons during the RC procedure (Liu and Au-Yeung, 2014). In addition, MEP latency is defined as the time from the delivery of TMS stimuli to MEP onset and reflects the conduction velocity of a motor efferent pathway. Compared with MEP amplitude, MEP latency has a lower level of variation (Kiers et al., 1993). Other studies on the lower limbs have demonstrated that MT and MEP latency for the TA muscles of healthy participants (Cacchio et al., 2009) or TA (Cacchio et al., 2011; Beaulieu et al., 2017b) and quadriceps muscles (Wheaton et al., 2009) of patients with stroke provide more reliable measurements than MEP amplitude. The present study showed that the ICCs for rMT and MEP latency exceeded 0.90, revealing excellent repeatability in the within-day or between-day comparison. Compared with rMT and MEP latency, the relatively lower ICCs of RC slope and pMEP indicated large variations in these two parameters, especially between-day reliability. This result was coherent with Cacchio’s study, which indicated excellent reliability for rMT (ICC = 0.98 and 0.97 for within-day and between-day reliability, respectively) and MEP latency (ICC = 0.93 and 0.92 for within-day and between-day, respectively) and good reliability for RC slope (ICC = 0.79 and 0.78 for within-day and between-day, respectively; Cacchio et al., 2009).

Variability in an MEP-related parameter can be attributed to intrinsic factors, such as physiological fluctuations in the excitability of cortical pyramidal neurons and spinal alpha motor neurons (Kiers et al., 1993; Darling et al., 2006). It is also related to other external factors, such as age, physiological status of participants, attention level, coil positioning, and assessment methodology (Ridding and Ziemann, 2010; Bhandari et al., 2016). In healthy participants, Cacchio et al. presented higher rMT (62.25% and 63.02% maximum stimulator output for two test-retest sessions respectively) and longer latency (32.20 and 32.44 ms for two test-retest sessions respectively) for TA muscle than those obtained in the present study (Cacchio et al., 2009). This inconsistency may be due to differences in equipment used between the two studies in terms of coil types, pulse waveform, current direction, and absolute magnetic output intensity of the stimulator. Cacchio’s group conducted the measurement with a circular coil, whereas the present study used a double-cone coil. Another reason may be the different characteristics of participants, such as age and height, in the two studies. Notably, the participants (mean age: 23.05 years) recruited in the present study were younger than those recruited in Cacchio’s study (mean age: 44.8 years), and whether the heights of the participants in the two studies varied is unknown.

The excitability of the motor cortex fluctuates with the level of awakening and attention of participants and may be observed by long-term studies (Classen et al., 1998). The fatigue and discomfort of participants are significantly related to the increment of the duration of assessment using TMS (van de Ruit et al., 2015). Thus, long-term testing will reduce participants’ compliance (Mead et al., 2012), and determining the minimum number of stimuli for TMS-induced outcome measures is reasonable. On the premise of ensuring accuracy, our study reduced the time cost and addressed these unfavorable factors during the assessment of the corticomotor excitability of the lower limb with TMS.

As predicted, when more TMS stimuli were provided, high ICCs and low SEM and MDD_95_ values for the RC slope and pMEP amplitude were found. In an assessment tool in clinical practice, the minimum acceptable value of ICC is 0.7 (Nunnally and Bernstein, 1994; Shieh, 2016; Matheson, 2019). Our results showed no obvious fluctuation in ICCs for the RC slope, pMEP amplitude, and MEP latency induced by different quantities of stimuli (3–10) when the assessments were conducted on the same day. Thus, three TMS pulses were suitable for the three TMS-related parameters, and adequate repeatability of different assessment sessions on the same day was observed. However, this inference should be treated conservatively in research and clinical practice, especially the inference about pMEP amplitude. The pMEP measured in the present study was not the simple MEP induced by a fixed TMS intensity. Rather, it was the maximum averaged MEP that appears on the RC curve. Second, the three stimuli only achieved the minimum acceptable degree of reliability for these three outcomes measured on the same day. Thus, a larger number of stimuli is recommended if time consumption and participants’ conditions are considered. On the between-day comparison, MEP latency showed excellent ICC even when three stimuli were used in the measurement protocol. TMS is a reliable measurement tool only if at least seven or nine stimuli are used in the assessment of the RC slope and pMEP amplitude, respectively. This result was similar to those of previous studies. Lewis et al. examined the reliability of the amplitude and area of MEP on the soleus muscle in healthy participants (Lewis et al., 2014). The results demonstrated that the between-day reliability was acceptable for healthy participants with an ICC value at a mean MEP amplitude of 0.71 and area of 0.85 when six pulses were used as stimuli (Lewis et al., 2014). By contrast, Cuypers’s study revealed that MEP amplitude averaged on eight consecutive stimuli is sufficient to enter the confidence interval for the first dorsal interosseous muscle in healthy individuals (Cuypers et al., 2014). The recent systematic review and meta-analysis of Cavaleri showed that the reliability of MEP amplitude on healthy participants is influenced by participants’ characteristics, target muscle, interval time, and coil type (Cavaleri et al., 2017). Their results have explained why the number of stimuli for obtaining a reliable MEP-related outcome measure in different studies are inconsistent. Therefore, our findings are only applicable to the corticomotor excitability measurement with double-cone coil for TA muscle in young healthy adults.

Our results should be interpreted with caution because of some limitations. Although the sample size of this study was within the range of other TMS reliability studies (Beaulieu et al., 2017a), the number of participants was relatively small with referring to previous studies which reported that more than 50 participants are suitable for reliability examination (Hopkins, 2000; Terwee et al., 2007). Moreover, the TMS-related parameters assessed in the present study were only using single-pulse TMS. Thus, whether the parameters examined with paired-pulse TMS are reliable is unclear. Although the conventional hand-held system of TMS remains the most widely used in clinical assessment, it cannot constantly localize hotspots in a longitudinal measurement and enable coil position tracking during a measurement session in contrast to the automatic devices such as navigated TMS and robot TMS coil holding arm (Sparing et al., 2008; Fleming et al., 2012). In addition, implementation of the closed-loop trigger strategy of phase-locked EEG-TMS can reduce the variability of MEP size caused by the brain oscillating rhythms (Zrenner et al., 2018). Hence, the reliability of TMS-induced corticomotor excitability measurements using new devices like navigated systems, robot coil arm and TMS-EEG for heterogeneous patient populations in large samples should be investigated further.

## Conclusion

Although our research is not entirely innovative in methods, it has provided novel insights into the reliability of TMS-related measurements for the lower limb in healthy participants. Determining the minimum number of stimuli provides an evidence-based meliorative protocol for assessing the corticomotor excitability of the lower limb. In addition, the reported SEM and MDD_95_ values of the four TMS measurements should be used as references for evaluating the effects of clinical trials involving people without neurological pathology.

## Data Availability Statement

The raw data supporting the conclusions of this article will be made available by the authors, without undue reservation.

## Ethics Statement

The studies involving human participants were reviewed and approved and the study was approved by the Research Ethics Committee of the JORU Rehabilitation Hospital (No. 20210325B02, ChiCTR2100045141). The patients/participants provided their written informed consent to participate in this study. Written informed consent was obtained from the individual(s) for the publication of any potentially identifiable images or data included in this article.

## Author Contributions

HL and LW designed the whole study. HL, LZ, and BS analyzed the data, and wrote the manuscript together. YJ, QS, DL, FG, and JW performed the study. YJ, GH, and YC participated in the collation of data. All authors contributed to the article and approved the submitted version.

## Funding

This study was supported by the Major Scientific Research Project of Wuxi Health Committee (Z202013), Top Talent Support Program for Young and Middle-aged People of Wuxi Health Committee (HB2020079), Wuxi “Taihu Talent Plan” medical and health high-level talents project (WXTTP2020008), Jiangsu Geriatrics Clinical Technology Application Research Project (LR2021040), Scientific and Technological Development Fund from Wuxi Science and Technology Bureau (Y20212008), and Clinical Teaching Base Scientific Research Development Project from Jiangsu Vocational College of Medicine.
